# Goiter and Laryngeal Sensory Neuropathy

**DOI:** 10.1155/2013/765265

**Published:** 2013-05-29

**Authors:** Abdul Latif Hamdan, Jad Jabour, Sami T. Azar

**Affiliations:** ^1^Department of Otolaryngology-Head & Neck Surgery, American University of Beirut Medical Center, P.O. Box 110-236, Beirut, Lebanon; ^2^Department of Internal Medicine, Division of Endocrinology and Metabolism, American University of Beirut Medical Center, P.O. Box 110-236, Beirut, Lebanon

## Abstract

*Objective*. Examining the prevalence of laryngeal sensory neuropathy (LSN) in goiter patients versus a control group. *Study Design*. Cross-sectional study. *Methods*. 33 Goiter patients were enrolled versus 25 age-matched controls. TSH levels, size of thyroid gland, and presence or absence of thyroid nodules were reported. Subjects were asked about the presence or absence of any of the following symptoms: cough, globus pharyngeus, and/or throat clearing that persistented for more than 6 weeks. The presence of one or more of these symptoms for at least six weeks in the absence of LPRD, allergy, asthma, ACE inhibitor intake, and psychogenic disorder was defined as LSN. *Results*. For goitrous patients mean age (years) was (41.73 ± 9.47) versus (37.44 ± 10.89) for controls. 82% goitrous patients had known nodules and 27% carried a simultaneous diagnosis of hypothyroidism. Among those with documented size (61%), mean total thyroid volume was 26.996 ± 14.852 cm^3^, with a range from 9.430 to 67.022 cm^3^. The overall prevalence of LSN among goitrous patients was 42% versus 12% among controls (*P* = 0.0187). There was no correlation between LSN, size of thyroid gland, and TSH level. *Conclusion*. The prevalence of LSN in goitrous patients is significantly higher than that in a nongoitrous population.

## 1. Introduction

Goiter is the most prevalent endocrine condition in the world affecting over 500 million with prevalence rates reaching up to 30% [1, 2]. It is believed to result from an interaction between genetics and environmental factors, namely, iodine deficiency. It has long been established that adequate iodine uptake is crucial, as low iodine levels lead to hypothyroidism which in turn increases blood levels of thyroid stimulating hormone (TSH) resulting in glandular hypertrophy [3]. Thus, certain conditions that exacerbate iodine deficiency, such as smoking or increased parity, can be considered risk factors for goiter [4]. While the exact contribution of each factor remains unclear, genetic predisposition to goiter also plays a crucial role in goitrogenesis [5]. 

Patients with goiter may complain of cosmetic disfigurement attributed to the visibly enlarged thyroid gland, of symptoms of hyper or hypothyroidism secondary to the altered levels of thyroid hormones, and last but not the least of compressive neck symptoms. These include respiratory discomfort, stridor, and change in voice quality, globus pharyngeus, dysphagia, and others. The neck and throat symptoms in patients with goiter have been invariably attributed to the glandular hypertrophy of the thyroid and its mass effect on the laryngotracheal framework. The enlarged gland may impede the movement of the larynx and trachea and thus interfere with the basic functions of the larynx, namely, phonation and swallowing. Compression and invasion of the laryngotracheal complex may also result in airway symptoms with narrowing of the lumen and or impaired mobility of the vocal fold with or without recurrent laryngeal nerve neuropathy [1]. 

No previous study has examined the possible presence of laryngeal sensory neuropathy (LSN) in patients with goiter despite the fact that LSN is a confounding etiology for many of the throat and pharyngeal symptoms in goitrous patients. The presence of globus pharyngeus, cough, throat discomfort, and change in voice quality in patients with goiter might be the clinical manifestation of other diseases beside goiter. [6, 7]. Laryngeal sensory neuropathy is usually suspected when other etiologies have been ruled out and/or when positive response to a neuromodulator is displayed. It is considered in the differential diagnosis of chronic cough (longer than 6 weeks), when conditions such as asthma, pneumonia, bronchitis, LPR, or ACE inhibitor adverse reactions have been ruled out [7]. The presence of throat discomfort of acute onset in addition to cough should allude more to the etiological role of laryngeal sensory neuropathy [8]. What helps to confirm the diagnosis of LSN is the response to neuromodulating agents such as pregabalin and gabapentin [7]. In a retrospective chart review of 12 patients prescribed pregabalin for symptoms of LSN, the mean treatment chief complaint symptom decreased from 3.9 to 1.2 after a one-month treatment with pregabalin.

The purpose of this study is to examine the prevalence of LSN in a cross-section of goiter patients compared to a control group. Improved knowledge of the prevalence of LSN status in patients with goiter may lead to better management of symptoms previously attributed to the mass effect of thyroid gland alone. Patients with goiter and laryngopharyngeal symptoms due to LSN may be treated with neuromdulators. The resolution of these symptoms may spare goitrous patients the need for surgical intervention.

The hypothesis of the study is that the prevalence of LSN is significantly higher in a population of goitrous patients compared to that in nongoitrous controls.

## 2. Methods

Thirty-Three consecutive patients with goiter were recruited over a two-month period at a private endocrinology clinic at the American University of Beirut Medical Center. All goitrous patients between the ages of 18–65 were informed of a study taking place in the adjacent “Hamdan Voice Unit” regarding the possible association of goiter with laryngeal sensory neuropathy. None of the patients with goiter refused to participate in the study. The diagnosis of goiter was based on clinical and/or radiological examination using ultrasound. Thirty-three patients enrolled in the study and signed a consent form approved by the Institutional Review Board at the American University of Beirut. During the same period, twenty-five age-matched controls were recruited by word of mouth. Exclusion criteria included history of laryngeal manipulation or current upper respiratory infection symptoms. 

Demographic data included age, gender, smoking status, laryngopharyngeal reflux disease, and allergy. The presence of laryngopharyngeal reflux disease was based on a reflux symptom Index above 9 using the reflux symptom index designed by Belafsky et al. [9]. The presence of allergy was confirmed using a validated questionnaire developed by Bauchau et al. [10]. 

For goiter patients, the following parameters were also reported: thyroid hormonal level, size of the thyroid gland, and presence or absence of thyroid nodules. The thyroid hormonal level was determined using their last Thyroid Stimulating Hormone test (TSH). A range between 0.27 and 4.20 microunit/mL was considered as normal. Findings from the most recent ultrasound were used to establish the size of the thyroid. The presence of thyroid nodule was determined on either clinical or radiological examination.

Subjects were asked about the presence or absence of any of the following symptoms: cough, globus pharyngeus, and or throat clearing that was persistent for more than 6 weeks. The presence of one or more of these symptoms for at least six weeks in the absence of laryngopharyngeal reflux disease, allergy, asthma, ACE inhibitor intake, and psychogenic disorder was defined as laryngeal sensory neuropathy based on the definition by Halum et al. [7]. Patients were considered to have no laryngopharyngeal disease when the reflux symptom index was less than nine. 

Simple descriptive analysis was used to determine the prevalence of vocal symptoms suggestive of LSN in this cross-sectional sample of goiter patients. The Fisher exact test was used to make comparisons between goiter patients and controls and between goitrous patients with and without hypothyroidism. The Pearson correlation coefficient was calculated to test for associations between the presence of LSN and goiter size.

## 3. Results

### 3.1. Demographic Data

 A total of 33 patients with goiter and 25 controls with no diagnosis of goiter were enrolled in the study. The mean age was 41.73 ± 9.47 for goitrous patients and 37.44 ± 10.89 for controls. The prevalence of smoking was 27% (9/33) among goitrous patients and 20% (5/25) among controls (*P* = 0.5551). The prevalence of allergies was 12% (4/33) among cases and 32% (8/25) among controls.

 Among goitrous patients, 82% (27/33) had known nodules. Twenty-seven percent (9/27) carried a simultaneous diagnosis of hypothyroidism; none had hyperthyroidism. For 61% (20/33), a documented thyroid size based on ultrasound performed within the last year was available. Among those with documented size, mean total thyroid volume was 26.996 ± 14.852 cm^3^, with a range from 9.430 to 67.022 cm^3^ (Table 1).

### 3.2. Prevalence of LSN in Patients with Goiter and Controls

The overall prevalence of LSN determined by the aforementioned criteria among goitrous patients was 42% (14/33). The prevalence among controls was 12% (3/25). The difference in proportions was statistically significant (*P* = 0.0187) (Table 2). 

### 3.3. Correlation between Laryngeal Sensory Neuropathy, Size of Thyroid Gland, and Thyroid Hormonal Level

Only twenty patients with goiter had documented ultrasound measurements of their thyroid gland. Only eight of these twenty subjects had LSN. Those with LSN had a mean thyroid volume of 24.126 ± 19.834 cm^3^, while those without LSN had a mean thyroid volume of 27.629 ± 11.209 cm^3^ (*P* = 0.5062). The calculated Pearson correlation coefficient was *r* = −0.118, giving a two-sided *P* value of 0.6188 (Table 3).

The prevalence of LSN among cases with simultaneous diagnosis of hypothyroidism was 44% (4/9), compared to 42% (10/24) among those without hypothyroidism (*P* = 1.000). There was no correlation between LSN, size of the gland, and hypothyroidism.

## 4. Discussion

 Chronic refractory cough is present in roughly 31% of the general population [10], attributed to a wide variety of conditions. Though relatively rare, laryngeal sensory neuropathy has fairly recently gained attention as a potential cause of not only chronic cough, but also throat discomfort and possibly dysphagia and dysphonia [7, 11]. It is thought to be the result of a decrease in the laryngeal sensory threshold leading to a variety of abnormal laryngeal behavior. These include abnormal glottic closure reflexes, intractable cough, throat clearing, impaired mobility of the vocal folds and vocal fold paralysis [8, 12, 13]. The diagnosis of laryngeal sensory neuropathy is that of exclusion after having excluded the aforementioned confounding diseases, namely; reflux, allergy, asthma, ACE inhibitor intake, and psychogenic disorders. Other diagnostic tests include fiberoptic laryngeal sensory testing through the repeated application of air puffs to elicit glottic closure reflex and using surface evoked laryngeal sensory action potential evaluation [11, 14]. 

 In view of the overlap in the laryngopharyngeal symptoms of patients with LSN and patients with goiter, the authors of this paper have been intrigued to examine the prevalence of LSN in patients with goiter. The hypothesis is that LSN is more prevalent in patients with goiter compared to controls. Fiorentino et al. had made a similar argument in relation to LPR pointing out other potential explanations for the local neck symptoms often experienced by goitrous patients and normally attributed solely to the goiter [6]. The hypothesis for the increased prevalence of LSN in the population of this study can largely be based on one or many of the suggested mechanisms for LSN previously described in the literature. Though the exact pathogenesis of LSN in general has not been elucidated, several theories have been suggested. These include viral infections, metabolic changes, and insult to either the recurrent laryngeal nerve or superior laryngeal nerve with subsequent change in the firing threshold [7]. Because LSN is thought to occur secondary to mechanical damage to nerves [7] and such damage can be associated with glandular hypertrophy [1], it is reasonable to expect a higher prevalence of LSN in patients with goiter. In view of the proximity of the thyroid gland to the laryngeal framework, hypertrophy of the thyroid gland may induce stretching of the recurrent laryngeal nerve, compression, reduction in its blood supply, perineural inflammation and fibrosis, and or simply direct invasion of the nerve [15]. All of the suggested mechanisms may result in damage to the laryngeal innervations with subsequent increase in laryngopharyngeal symptoms. 

Indeed, the results of our study supported the hypothesis that the prevalence of LSN in a population of goitrous patients is significantly higher than that in a population of nongoitrous controls. Nearly half (42%) of goitrous patients in this study had LSN compared to only 12% in the control group. The prevalence of smoking in both subgroups, goitrous patients with LSN and controls with LSN, was similar (35.7% versus 33.3%, resp.). What is surprising on the other hand is the lack of correlation between the size of the gland and LSN in our study. The results of the Pearson correlation analysis suggest that there is no significant association between thyroid volume and prevalence of LSN (*r* = −0.118; *P* = 0.6118). The lack of correlation in our study might be attributed to the small size sample and the relatively moderate size of the gland in our subject population (mean of 26.996 ± 14.852 cm^3^). While a correlation with thyroid size might be expected based on the proposed mechanism of association between goiter and LSN previously mentioned, further studies would be needed to elucidate this relationship. 

 Another possible explanation for the LSN is metabolic damage. Common example is sensory neuropathy in patients with diabetes mellitus. Poor glycemic control is known to result in nerve degeneration with resultant demylination and poor nerve conduction [16]. Subsequently patients with diabetic neuropathy experience symptoms of numbness, paresthesia, and pain. Similarly, thyroid diseases have been reported to cause signs and symptoms of neuromuscular dysfunction [17–20]. Most often reported clinical features are proximal muscle weakness, mononeuropathy, and sensorimotor polyneuropathy. In a large case-control cross-sectional study on 40 patients with hypothyroidism, the majority had carpel tunnel syndrome [18]. In another prospective study by El-Salem and Ammari on twenty-three neurologically asymptomatic patients with primary hypothyroidism, nerve conduction studies revealed that almost half of the subjects had some abnormality predominantly of the motor demylinating pattern, and nondisfigurative myopathic changes were seen in 74% of the patients most commonly affecting the deltoid [19]. In corroboration with these results, Duyff et al., in his evaluation of patients newly diagnosed with hypo- and hyperthyroidism, indicated that neuromuscular symptoms and signs were commonly present and that 40% and 20% of hypothyroid and hyperthyroid patients had sensory signs of a sensorimotor axonal neuropathy [20]. 

In this study, it did not appear that hypothyroidism had an independent effect on LSN, as subgroup analysis between goitrous patients with and without hypothyroidism revealed no significant difference in prevalence of LSN (*P* = 1.000). This lack of correlation between hypothyroidism and LSN is not commensurate with the previous reports on the neuromuscular and sensory motor status in patients with hypothyroidism. This can be attributed to the small sample size and the lack of any objective measures to detect subclinical neuromuscular manifestations.

There are two main limitations to this study. The first is the relatively small sample size and the second is the lack of objective diagnostic tests for LSN employed. For instance, LSN can be associated with alterations in surface evoked laryngeal sensory action potential (SELSAP) waveforms [15]. Alternatively, fiberoptic endoscopy can be used to evaluate laryngeal sensation [17]. Nonetheless, LSN remains primarily a clinical diagnosis, and thus clinical criteria were used in this study.

To the authors' knowledge, this is the first paper to explicitly examine the prevalence of LSN in goitrous patients. A more extensive study, with similar methodologies, could be done to corroborate the findings presented here. The highly significant *P* value (0.0187) strongly suggests that a true difference in LSN prevalence exists between goitrous and nongoitrous patients. Consequently, it may be worthwhile to consider a neuromodulating agent to relieve the patient's symptoms, before concluding that they are a direct result of the goiter and would be relieved by surgery. Further research would need to be undertaken on this question before proposing any official recommendations.

## 5. Conclusion

The prevalence of laryngeal sensory neuropathy (LSN) in a population of goitrous patients is significantly higher than that in a nongoitrous population. Symptoms of persistent cough, throat clearing, dysphonia, or globus pharyngeus in those with goiter may be attributable to LSN, rather than the goiter itself. Physicians should be aware of how this possible etiology could alter the optimal management approach.

## Figures and Tables

**Table 1:** Demographic data for cases and controls, including prevalence of smoking, reflux, allergies, and concurrent endocrine conditions.

	Cases	Controls
Total number (*n*)	33	25
Mean Age ± SD	41.73 ± 9.47	37.44 ± 10.89
Gender (% males)	6	40
Smoking (%)	27	20
Reflux (%)	12	12
Allergy (%)	12	32
Mean thyroid size (cm^3^)	26.996 ± 14.852	
(Range)	9.430–67.022	
Thyroid stimulating hormonal level		
Hypothyroidism (%)	27	0
Hyperthyroidism (%)	3	0
Euthyroid (%)	70	0

Thyroid nodules (%)	82	—

**Table 2:** Prevalence of Laryngeal Sensory Neuropathy (LSN) in cases and controls.

	Cases	Controls	*P*-value
LSN (%)	42	12	0.0187

**Table 3:** Results of Fisher exact test and Pearson correlation test for association between thyroid size and LSN prevalence, no significant *P* values.

Mean thyroid size (cm^3^)	Goitrous pts with LSN	Goitrous patients without LSN	*P* value
	24.126 ± 19.834	27.629 ± 11.209	0.5062
Pearson correlation coeff. (*r*)	−0.118		
2-side *P* value	0.6118		
